# Use of Novel 6.3 Fr Ureteroscope in Endoscopic Combined Intrarenal Surgery (ECIRS): Comparative Experience with Conventional Ureteroscopes

**DOI:** 10.3390/jcm15093537

**Published:** 2026-05-06

**Authors:** Theodoros Spinos, Vasileios Tatanis, Angelis Peteinaris, Fani Moultsia, Dimitrios Diamantopoulos Kogkas, Paraskevi Katsakiori, Vasiliki Tsekoura, Theofanis Vrettos, Evangelos Liatsikos, Panagiotis Kallidonis

**Affiliations:** 1 Department of Urology, University Hospital of Patras, 26504 Patras, Greece; thspinos@otenet.gr (T.S.); tatanisbas@gmail.com (V.T.); peteinarisaggelis@gmail.com (A.P.); moultsiafani@gmail.com (F.M.); dimitriskogkas97@gmail.com (D.D.K.); vkatsak@gmail.com (P.K.); vicktsek@yahoo.gr (V.T.); liatsikos@yahoo.com (E.L.); 2 Department of Anesthesiology, University Hospital of Patras, 26504 Patras, Greece; teovret@gmail.com; 3 Department of Urology, Medical University of Vienna, 1090 Vienna, Austria

**Keywords:** endourology, urolithiasis, ECIRS, miniaturization, 6.3 Fr

## Abstract

**Background/Objectives**: Recently, a 6.3 Fr single-use flexible ureteroscope (f-URS) was introduced to the market. The purpose of this pilot study is to present our experience with it during Endoscopic Combined Intrarenal Surgery (ECIRS) and to compare its performance with the conventional 7.5 Fr scope. **Methods**: For percutaneous access, renal puncture was performed in a nonpapillary approach. Regarding retrograde access, for the first group, a 7.5 Fr single-use f-URS was used, while for the second group, a 6.3 Fr single-use f-URS was utilized. Lithotripsy was primarily performed in an antegrade manner, using the Lithoclast Trilogy^®^. In cases where stones could not be reached with a nephroscope, retrograde lithotripsy was performed with either a Holmium:YAG laser or a Thulium Fiber Laser. **Results**: In total, 45 patients were included. Of these, 23 patients underwent ECIRS with the 6.3 Fr f-URS and 22 with the 7.5 Fr f-URS. The mean operative time, fluoroscopy time and lasing time were 59.5 ± 5.6 min, 139.7 ± 14.2 s and 18.4 ± 2.7 min in the 6.3 Fr group and 57.1 ± 3.9 min, 133.8 ± 29.7 s and 18.6 ± 1.9 min in the 7.5 Fr group, respectively. Two patients in the 6.3 Fr group and three patients in the 7.5 Fr group experienced Grade II complications. Stone-free rates were 91.3% in the 6.3 Fr group versus 86.4% in the 7.5 Fr group. **Conclusions**: The use of a 6.3 Fr f-URS during ECIRS is potentially a feasible, safe and efficient approach. Both the 6.3 Fr and 7.5 Fr scopes were associated with comparable outcomes during ECIRS. Additional studies are needed so as to draw safer conclusions.

## 1. Introduction

Endoscopic Combined Intrarenal Surgery (ECIRS) refers to the combination of retrograde and antegrade approaches for the management of large or complex intrarenal stones [1]. Although the term was first used in 2008 by Scoffone et al. [2], its origins date back to the 1990s, when Dr. Gaspar Ibarluzea in Spain utilized both a nephroscope and a ureteroscope to treat complex stones in the Valdivia supine position [3]. Flexible ureteroscopes (f-URS) are essential components of modern ECIRS procedures [4]. Interestingly, single-use f-URS of different sizes achieve comparable outcomes to conventional reusable scopes, although their cost-effectiveness and environmental impact remain under investigation [5]. Recently, a 6.3 Fr single-use f-URS was introduced to the market, and it still represents the thinnest scope currently available. This ureteroscope may be particularly advantageous for pediatric patients and patients with complex urinary tract anatomy [6]. However, it is also potentially associated with certain drawbacks, including a narrower working channel and increased susceptibility to bending [6]. The purpose of this pilot study is to present our experience with the new 6.3 Fr ureteroscope during ECIRS, including aspects such as articulation and torque performance, and to compare its performance with the conventional 7.5 Fr scope, which was routinely used prior to its introduction and continues to be employed in many cases.

## 2. Materials and Methods

### 2.1. Operative Technique and Equipment

**Figure 1:**
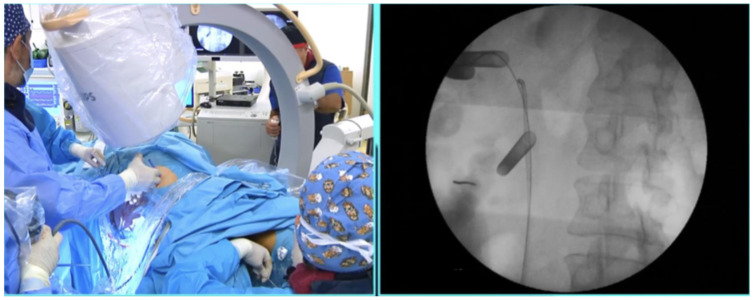
Operating room setup and patient positioning. Fluoroscopy demonstrates both retrograde and antegrade accesses used during ECIRS.

All surgeries were carried out under general anesthesia. A ureteral stent (double-J) was placed preoperatively in all patients, one week prior to the surgery (pre-stented approach). Almost all patients underwent open-ended ureteral catheter placement in the lithotomy position with a rigid cystoscope. The ureteral catheter was advanced to the upper calyces, following a retrograde pyelogram, and was secured to a Foley catheter (14 Fr in men and 16 Fr in women) with sutures. The patients were then repositioned in the prone position. The split-leg modification was used to allow for easy retrograde access in male patients (Figure 1). Regarding the percutaneous access for percutaneous nephrolithotomy (PCNL), a renal puncture was performed in a nonpapillary approach, usually near the lower or middle calyces. The upper calyces were avoided in an attempt to avoid thoracic complications, but sometimes this was not feasible. Amplatz dilators were utilized for tract dilation (Amplatz renal dilator set, COOK Medical, Bloomington, IN, USA). For some patients, additional punctures (second or third) were deemed necessary so as to achieve optimal stone-free rates (SFRs). However, taking advantage of the ureteroscopic aid of the ECIRS, additional punctures were minimized to only selected cases with staghorn stones or complicated anatomies. Following the insertion of a 22 Fr sheath (mini-PCNL) using a one-step dilation method, an 18 Fr rigid nephroscope was passed through the sheath. Technical details regarding the puncture and the dilation times for this approach have been reported in an earlier work [7].

For retrograde intrarenal surgery (RIRS), a nitinol wire with a hydrophilic tip (Sensor, Boston Scientific, Marlborough, MA, USA) and a stiff guidewire (Amplatz Super Stiff, Boston Scientific) were inserted with the aid of a dual-lumen ureteral catheter. The first guidewire (Sensor) was inserted through the open-ended ureteral catheter. A meticulous exploration of the ureter and the pelvicalyceal system was then performed using a flexible ureterorenoscope. For the first group (Group A), a 7.5 Fr single-use EPU3033A f-URS (PUSEN Medical, Shenzhen, China) was used, while for the second group (Group B), a 6.3 Fr single-use HU30M f-URS (HugeMed Medical Technical Development Co., Ltd., Shenzhen, China) was utilized.

Lithotripsy was primarily performed in an antegrade manner, using the Lithoclast Trilogy^®^ (EMS Medical, Nyon, Switzerland). In cases where the nephroscope could not reach the stones despite optimal bending, taking advantage of the ECIRS approach, retrograde lithotripsy was carried out. Either a Holmium:YAG laser (Cyber Ho 150^®^, Quanta System, Samarate, Italy) or a Thulium Fiber Laser (TFL) (LaserClast Thulium Power, EMS Medical, Nyon, Switzerland) was utilized. For both technologies, laser energy was set between 1 and 2 J with frequencies ranging from 30 to 60 Hz. A high-power “self-popping” technique was used for retrograde lithotripsy, which has been described previously [8]. In some cases, stones or fragments were repositioned with the ureteroscope or with nitinol baskets (“pass the ball” maneuver) for later removal from the antegrade access.

At the end of each procedure, a retrograde re-exploration of the pelvicalyceal system was performed in order to confirm the total absence of residual fragments. A double-J stent (6–8 Fr) and a balloon nephrostomy tube (16–18 Fr) for drainage were placed in all cases. The nephrostomy tubes were usually removed on the second or third postoperative day, given that patients were afebrile and that urine was clear. Double-J stents were normally removed two to four weeks after surgery. The intraoperative parameters collected for analysis included total operative time, fluoroscopy time and lasing time. Operative time was defined as the interval from puncture initiation to the placement of the drainage tube.

### 2.2. Inclusion and Exclusion Criteria

The following inclusion criteria were followed for the implementation of ECIRS: stones that were located in calyces that could not be reached with the nephroscope, patients with challenging pelvicalyceal system angles and anatomical variations (such as horseshoe kidneys, duplicated pelvicalyceal systems and malrotation) and finally stones located in areas where an additional access would be risky for thoracic complications (such as stones in upper calyces over the 11th rib). No specific exclusion criteria were applied, while all cases were unselected. Table 1 presents all inclusion criteria.

### 2.3. Statistical Analysis

Statistical analysis was performed with Statistical Package for the Social Sciences (SPSS) software, version 22.0 (IBM Corp., Armonk, NY, USA) [9]. Mean values and standard deviations were used to report continuous variables. The Kolmogorov–Smirnov test was utilized for the evaluation of the data normality distribution. The *t*-test was used for comparing continuous variables with a normal distribution, while non-normally distributed continuous variables were compared with the Mann–Whitney U test. Frequencies and percentages were utilized for reporting categorical variables, while the chi-square test was used for their comparison. A cutoff of a *p*-value < 0.05 was regarded to define statistical significance.

## 3. Results

A retrospective analysis of prospectively collected data was performed. A detailed comparison between the 7.5 Fr and 6.3 Fr ureteroscopes during ECIRS is provided in Table 2. In total, 45 patients were included in the current study. Of these, 23 patients underwent ECIRS with the 6.3 Fr f-URS (40 stones in total) and 22 with the 7.5 Fr f-URS (41 stones in total). The male-to-female ratio was 16/7 in the 6.3 Fr group and 12/10 in the 7.5 Fr group. The mean age and body mass index (BMI) were 52.2 ± 12.4 years old and 25.7 ± 3.7 kg/m^2^ in the 6.3 Fr group, compared to 53.9 ± 14.9 years and 24.6 ± 3.43 kg/m^2^ in the 7.5 Fr group. Anticoagulation therapy was reported in 39.1% of patients in the 6.3 Fr and 31.8% in the 7.5 Fr group. In all cases, treatment was discontinued two to three days prior to surgery. Regarding stone laterality, 26.1% of stones were right-sided and 73.9% were left-sided in the 6.3 Fr group, whereas 36.4% were right-sided and 63.3% left-sided in the 7.5 Fr group. Stone location did not vary significantly between the two groups. For the 6.3 Fr group, 42.5% of the stones were located in the renal pelvis, 7.5% in the upper calyces, 10% in the middle calyces and 40% in the lower calyces. Likewise, in the 7.5 Fr group, 29.3% of the stones were located in the renal pelvis, 22% in the upper calyces, 2.4% in the middle calyces and 46.3% in the lower calyces. The mean stone density was 965.8 ± 187.3 Hounsfield Units (HU) in the 6.3 Fr group and 826.1 ± 143.3 HU in the 7.5 Fr group. This difference was statistically significant (*p* = 0.001). Mean stone burden was similar between the two groups (1969.3 ± 859.7 mm^3^ in the 6.3 Fr group versus 2073 ± 918.2 mm^3^ in the 7.5 Fr group).

Intraoperative and postoperative outcomes were also comparable between the two groups. The mean operative time, fluoroscopy time and lasing time were 59.5 ± 5.6 min, 139.7 ± 14.2 s and 18.4 ± 2.7 min in the 6.3 Fr group and 57.1 ± 3.9 min, 133.8 ± 29.7 s and 18.6 ± 1.9 min in the 7.5 Fr group, respectively. The mean length of hospitalization did not differ significantly between the two groups (4.09 ± 1.1 days in the 6.3 Fr group versus 4.4 ± 2 days in the 7.5 Fr group). Complications, classified according to the Clavien–Dindo Classification System, were comparable between the two groups [10]. Two patients in the 6.3 Fr group and three patients in the 7.5 Fr group experienced Grade II complications, consisting of fever or mild hematuria in all cases, all of which resolved with conservative management. No Grade III-IV complications were reported in either group. The mean hemoglobin decrease and creatinine change were not significantly different between groups (0.85 ± 0.58 g/dL and 0.17 ± 0.1 mg/dL in the 6.3 Fr group versus 0.85 ± 0.7 g/dL and 0.1 ± 0.06 mg/dL in the 7.5 Fr group). Finally, stone-free rates (SFRs) were comparable between the two groups (91.3% in the 6.3 Fr group versus 86.4% in the 7.5 Fr group). SFRs (stone fragments ≤ 2 mm) were determined 1 month postoperatively, either by performing a computed tomography scan (CT) of the kidneys, ureters and bladder (KUB) or by a combination of KUB ultrasound and KUB X-ray postoperatively.

## 4. Discussion

Miniaturization has become a defining trend in PCNL, with the development of mini-PCNL and ultra-mini-PCNL [11,12]. A similar evolution has been reported in RIRS, where progressively thinner f-URS are constantly released [13]. Until recently, 7.5 Fr single-use ureteroscopes were the thinnest available on the market. Their use has been associated with several benefits, and they have gained increased popularity in the management of pediatric stone disease [14,15]. Further miniaturization followed with the introduction of 6.3 Fr single-use f-URS, which still represents the thinnest scope currently available. The current study investigates whether adopting the thinnest available f-URS during ECIRS is a feasible, safe and efficient approach. The 6.3 Fr f-URS was associated with comparable outcomes with the 7.5 Fr f-URS, while no cases of undesirable scope bending or damage were reported.

Several applications of the novel 6.3 Fr f-URS have been reported. Ebner et al. presented their initial experience with the 6.3 Fr scope in 34 consecutive patients, supporting its feasibility and safety [16]. They also performed matched analyses with a 7.5 Fr f-URS, reporting that the 6.3 Fr f-URS was associated with shorter operative times [16]. Krajewski et al. performed a prospective randomized trial comparing the 6.3 Fr and 7.5 Fr f-URS during RIRS in 30 patients [17]. Both scopes demonstrated comparable performance and outcomes in their study, while image quality and maneuverability scores were also similar between the two groups [17]. Likewise, Geavlete et al. compared the 6.3 Fr and 7.5 Fr f-URS during RIRS in a prospective comparative study consisting of 40 patients [18]. Interestingly, the authors reported that the 6.3 Fr one was associated with significantly better SFRs and shorter operative times [18]. Ding et al. performed a randomized controlled trial (RCT) to compare the outcomes of 6.3 Fr and 7.5 Fr f-URS, including 70 patients with upper urinary tract stones [19]. Although most outcomes, including SFRs, were comparable between the two groups, the 6.3 Fr group was associated with shorter operative time, while this difference was statistically significant [19]. Alharran et al. summarized these finding and performed a systematic review and meta-analysis comparing the performance of 6.3 Fr and 7.5 Fr f-URS [20]. They concluded that both scopes had similar safety profiles, whereas the 6.3 Fr one was associated with shorter total operative times. Nevertheless, no differences in the SFRs between the two scopes were documented [20]. The authors highlighted that the findings of these studies should be interpreted cautiously, taking into consideration the methodological limitations of the included studies [20].

Ayyappan et al. evaluated the physical characteristics of seven f-URS and assessed their deflection capacity when used with a flexible and navigable suction access sheath (FANS) [20]. The 6.3 Fr f-URS (HugeMed) had the smallest diameter but also demonstrated the lowest irrigation flow rate (20 mL/min) [21]. Yuan et al. retrospectively compared the 6.3 Fr and 8.5 Fr f-URS in RIRS with FANSs of different diameters in 67 patients [22]. Although SFRs and complication rates were comparable between the two scopes, the 6.3 Fr scope was again associated with shorter operative times [22]. Menzies-Wilson et al. compared the irrigation fluid flow between different diameters of f-URS and FANSs in an ex vivo model [23]. They found that using an 11/13 Fr FANS and maintaining an intrarenal pressure (IRP) of ≤30 mmHg, irrigation at approximately 700 mmHg (~120 mL/min) was achievable with the 6.3 Fr f-URS [23].

The comparison of ECIRS with other treatment modalities for the management of large intrarenal stones has been thoroughly investigated in the literature. Perella et al. compared ECIRS and PCNL in their matched case–control retrospective comparative study including 165 patients with comparable baseline characteristics [24]. The authors emphasized that although both approaches were associated with similar complication rates, ECIRS demonstrated higher SFRs especially for large and complex kidney stones [24]. Likewise, Fonseka et al. performed a propensity score matched analysis to compare ECIRS and supine PCNL in 704 patients [25]. Interestingly, ECIRS was associated with higher SFRs, shorter hospitalization time and lower secondary puncture rates [25]. Although both techniques were associated with comparable complication and transfusion rates, ECIRS demonstrated longer total operative time [25]. Noah et al. compared ECIRS and prone PCNL in a randomized controlled trial comprising 60 patients [26]. The authors reported longer total operative time and a higher number of total punctures in the PCNL group, while the ECIRS group was associated with higher single-step SFRs [26]. Finally, Abdullatif et al. compared the two approaches in a systematic review and meta-analysis, reporting higher SFRs for ECIRS and comparable operative time, complication rates and blood loss [27].

RIRS is another routinely performed technique for large intrarenal stones. Kassem et al. compared RIRS and PCNL for the management of large intrarenal stones in a prospective randomized controlled trial [28]. They reported similar SFRs between the two groups with comparable mean stone size preoperatively [28]. However, RIRS was associated with shorter hospital stay and less postoperative pain and hemoglobin drop but also with higher total operative time and postoperative fever rates [28]. Kang et al. performed a systematic review and meta-analysis to compare RIRS and PCNL SFRs from data arising exclusively from randomized controlled trials, showing that PCNL was associated with better SFRs overall [29]. Finally, the advent of FANSs has significantly increased the efficacy of RIRS during the management of large renal stones and staghorn stones. Cacciatore et al. compared the efficacy of FANSs versus conventional ureteral access sheaths in an Italian multicenter study [30]. The authors reported that FANSs were associated with shorter total operative time, better SFRs and lower complication rates [30].

Certain limitations of the present study must be acknowledged. To begin with, no power analysis was performed, and the sample size was relatively small (n = 45 patients). However, this was a pilot study reporting our initial experience with the 6.3 Fr f-URS during ECIRS. Moreover, although the data were prospectively collected, the analysis itself was retrospective. Another important limitation is that stone density was higher in the 7.5 Fr group, which may represent a confounding factor. Although this difference was statistically significant, the absolute difference in mean values was modest (965.8 ± 187.3 HU in the 6.3 Fr group versus 826.1 ± 143.3 HU in the 7.5 Fr group). However, its potential impact on operative outcomes (particularly lasing time and SFR) should be recognized. Given that harder stones are generally more challenging to fragment, the comparable outcomes observed in the 6.3 Fr group could actually reflect a favorable bias toward that scope. Importantly, mean stone size was comparable between the two groups. Another important limitation is that the main lithotripsy in ECIRS was performed antegradely via the nephroscopic route using the Lithoclast Trilogy^®^ system. However, both the antegrade and retrograde components of ECIRS are important, as they attempt to access difficult areas. Therefore, it is difficult to adequately assess the clinical utility of the retrograde flexible ureteroscope component for lithotripsy, while the present comparison may mainly reflect overall ECIRS outcomes rather than the specific contribution of the flexible ureteroscope used for the retrograde lithotripsy. Furthermore, this was a pilot study, and the sample size was relatively small to allow for reliable multivariate analysis. For all patients undergoing ECIRS in the current study, a pre-stenting approach was followed, and thus the potential benefit of the 6.3 Fr scope to reduce the need for pre-stenting was not demonstrated. However, the main purpose of this study was to evaluate instruments with different diameters, as well as their ability in terms of articulation and torquing. Additionally, both TFL and Holmium:YAG lasers were used, while no data about the exact number of cases in which each laser type was used were available. Finally, SFRs were not homogenously reported, as both CT KUB and a combination of KUB ultrasound and KUB X-ray were utilized for their calculation. Moreover, this manuscript does not report any preoperative nephrolithometric complexity assessment, such as the Resorlu–Unsal Stone Score (RUSS) or similar systems [31].

## 5. Conclusions

The use of a 6.3 Fr f-URS during ECIRS is potentially a feasible, safe and efficient approach. Both the 6.3 Fr and 7.5 Fr scopes were associated with comparable outcomes during ECIRS, including SFRs and complication rates according to the Clavien–Dindo Classification System. No cases of undesirable scope bending or damage were reported in the 6.3 Fr group. Additional studies on larger populations and with better study designs, such as prospective and randomized trials, are needed so as to draw safer conclusions.

## Figures and Tables

**Table 1:** Inclusion criteria (indications for ECIRS).

Inclusion Criteria (Indications for ECIRS)	1. Stones that were located in calyces that could not be reached with the nephroscope
	2. Stones in patients with challenging pelvicalyceal system angles and anatomical variations (such as horseshoe kidneys, duplicated pelvicalyceal systems and malrotation)
	3. Stones located in areas where an additional access would be risky for thoracic complications (such as stones in upper calyces over the 11th rib)

**Table 2:** Outcomes of overall sample.

Variable	6.3 Fr	7.5 Fr	*p*-Value
Patients (*n*)	23	22	
Gender (%)			0.29
Male	69.6% (16/23)	54.5% (12/22)	
Female	30.4% (7/23)	45.5% (10/22)	
Age (mean, SD)	52.2 ± 12.4 years	53.9 ± 14.9 years	0.67
BMI (mean, SD)	25.7 ± 3.7 kg/m^2^	24.6 ± 3.43 kg/m^2^	0.29
Anticoagulants (%)	39.1% (9/23)	31.8% (7/22)	0.60
Stones (n)	40	41	
Laterality (%)			0.46
Right	26.1% (6/23)	36.4% (8/22)	
Left	73.9% (17/23)	63.6% (14/22)	
Location (%)			0.12
Pelvis	42.5% (17/40)	29.3% (12/41)	
Upper Calyx	7.5% (3/40)	22% (9/41)	
Middle Calyx	10% (4/40)	2.4% (1/41)	
Lower Calyx	40% (16/40)	46.3% (19/41)	
Hounsfield Units (mean, SD)	965.8 ± 187.3	826.1 ± 143.3	0.001 *
Burden (mean, SD)	1969.3 ± 859.7 mm^3^	2073 ± 918.2 mm^3^	0.7
Operative Time (mean, SD)	59.5 ± 5.6 min	57.1 ± 3.9 min	0.11
Fluoroscopy Time (mean, SD)	139.7 ± 14.2 s	133.8 ± 29.7 s	0.41
Lasing Time (mean, SD)	18.4 ± 2.7 min	18.6 ± 1.9 min	0.77
Hospitalization (mean, SD)	4.09 ± 1.1 days	4.4 ± 2 days	0.51
Hemoglobin Drop (mean, SD)	0.85 ± 0.58 g/dL	0.85 ± 0.7 g/dL	0.99
Creatinine Change (mean, SD)	0.17 ± 0.1 mg/dL	0.1 ± 0.06 mg/dL	0.18
Complications (Clavien–Dindo) (%)	9% (2/23)	13.6% (3/22)	0.6
Grade I	0	0	
Grade II	2	3	
Grade III	0	0	
Grade IV/V	0	0	
Follow-up Duration (mean, SD)	10.8 ± 1.3 months	10 ± 1.4 months	0.04 *
SFR (%)	91.3% (21/23)	86.4% (19/22)	0.59

BMI: Body mass index (kg/m^2^). SFR: Stone-free rate. * The difference is statistically significant.

## Data Availability

Data are available on request by the corresponding author.

## Abbreviations

The following abbreviations are used in this manuscript:

ECIRS	Endoscopic Combined Intrarenal Surgery
f-URS	Flexible Ureteroscope
TFL	Thulium Fiber Laser
PCNL	Percutaneous Nephrolithotomy
SFR	Stone-Free Rate
RIRS	Retrograde Intrarenal Surgery
SPSS	Statistical Package for the Social Sciences
BMI	Body Mass Index
HU	Hounsfield Units
CT	Computed Tomography
KUB	Kidneys, Ureters and Bladder
FANS	Flexible and Navigable Suction Access Sheath
